# Examination of Congruity between Subjective and Objective Working Memory in Veterans with Mild TBI and Relation to Psychiatric Symptoms and Childhood Trauma

**DOI:** 10.3390/bs14100932

**Published:** 2024-10-11

**Authors:** Lisa N. Cruz, Nicole C. Walker, Sonia S. Rehman, M. Windy McNerney, Michelle R. Madore

**Affiliations:** 1 Mental Illness Research, Education, and Clinical Center, VA Palo Alto Healthcare System, Palo Alto, CA 94304, USA; lisacruz@stanford.edu (L.N.C.);; 2 Department of Psychiatry and Behavioral Sciences, Stanford University School of Medicine, Stanford, CA 94305, USA; 3 Chesapeake Center, Bethesda, MD 20817, USA

**Keywords:** subjective cognitive concerns, traumatic brain injury, mild TBI, veterans, working memory, executive functioning

## Abstract

Objectives: There is conflicting evidence regarding congruence between subjective cognitive decline and objective cognitive performance for individuals with a history of mild traumatic brain injury (mTBI). The current study investigated the congruity between subjective and objective cognition, particularly working memory, among veterans with an mTBI history, accounting for post-traumatic stress disorder (PTSD) and childhood trauma. Methods: Participants included 35 veterans with a history of mTBI sustained during deployment. Participants completed measures of subjective [i.e., Behavioral Inventory Rating of Executive Functioning (BRIEF)] and objective working memory (i.e., WAIS-IV working memory index). Congruity between subjective and objective working memory was examined using linear regression. Bonferroni-corrected correlations were run to explore relationships among working memory, psychiatric symptoms, mTBI severity, and childhood trauma. Results: Among Veterans with mTBI, subjective working memory and objective working memory performance were not significantly related (*p* > 0.05); however, the overall model was significant (*p* < 0.0001), and childhood trauma was a notable predictor (*p* = 0.02). Greater PTSD, depression, and sleep symptoms were significantly related to increased subjective working memory concerns, even after Bonferroni adjustments (*ps* < 0.0001). Better objective working memory was significantly related to a fewer number of childhood traumatic events; however, this did not sustain corrections. The majority of individuals (67%) endorsed significant working memory complaints, despite objectively performing within normal limits (within 1 SD and above). Conclusions: Subjective-objective working memory congruity among veterans with mTBI was limited. Subjective, but not objective, working memory concerns were associated with greater PTSD, depression, and sleep symptoms. Childhood trauma was a notable factor that contributed to both subjective and objective cognitive concerns. There remains clinical value in assessing subjective cognitive concerns given the strong relationships with psychiatric problems and, hence, a focus for intervention.

## 1. Introduction

Following their service in Operation Enduring Freedom, Operation Freedom’s Sentinel, and the Iraq War, veterans often report high rates of mild traumatic brain injury (mTBI) [1,2,3,4] as well as post-traumatic stress disorder (PTSD) [5,6]. In the context of experiencing a traumatic brain injury, psychiatric trauma symptoms may naturally come hand in hand [7], and symptom overlap between mTBI and PTSD may make differential diagnosis challenging. mTBI and PTSD can trigger overlapping cognitive, emotional, and physical symptoms [8], which can subsequently negatively impact psychosocial and health functioning [2,9,10,11]. Furthermore, factors such as childhood trauma add a layer of complexity that is not typically taken into consideration. The literature indicates that experiencing early trauma (e.g., childhood trauma) is strongly associated with service-related PTSD [12,13], even when accounting for combat exposure, which may further compromise functioning [14]. HPA-axis dysregulation due to early life trauma may hamper emotional learning and emotion regulation, disrupting an individual’s ability to acquire adequate coping skills to deal with future life stressors [15,16].

Accumulating data have demonstrated that a history of mTBI [17,18,19,20,21], PTSD [22,23], and childhood trauma [24,25] is independently associated with an increased risk of dementia, regardless of the etiology. Given that 8.1 million (or almost 50%) of veterans are currently aged 65 or older [26], there is value in forecasting the development of clinically relevant age-related conditions, such as Alzheimer’s disease and other dementias. Subjective cognitive concerns have been theorized to be one of the first symptoms of many dementias and have been associated with an increased risk of future cognitive decline [27,28]. Subjective cognitive concerns may represent a prodromal state of mild cognitive impairment [29,30,31] as well as an early sign of dementia [32]. However, the diagnostic validity of subjective cognitive concerns has also been questioned given mixed evidence [33,34]. In terms of mTBI and dementia risk, a dose-response relationship has been demonstrated, with greater dementia risk conferred by more severe or multiple brain injuries (e.g., mTBI with loss of consciousness (LOC) confers greater dementia risk relative to mTBI without LOC) [19]. Based on two large cohort studies of Afghanistan and Iraq veterans, individuals with comorbid mTBI, depression, and PTSD reported the greatest amount of cognitive difficulties and were found to be at the highest risk of unemployment relative to veterans without the three mentioned comorbidities [35,36].

Regardless of the population or clinical disorder assessed, there is conflicting evidence regarding the concordance between subjective cognitive decline and objective cognitive performance [37,38,39,40]. Among adults with self-reported memory concerns, a meta-analysis consisting of 50 studies and 58,778 participants found a small but significant association between subjective and objective memory concerns [41]. More specifically, increased subjective memory complaints were correlated with worse objective cognitive performance (r = −0.13, *p* < 0.001). In another meta-analysis of normatively aging older adults with 53 studies and 20,139 participants, a small association between subjective and objective memory was found [42]. Across the studies, correlations ranged from −0.29 to 0.41, and using a VC model, they found an unweighted mean effect size of r = 0.062, SE = 0.014. In an additional moderator analysis, Crumley and Stetler [42] found that age, years of education, gender, depression symptoms, length and type of subjective memory complaint, and type of objective memory impairment were significantly associated with effect size.

Among veterans with either TBI or mTBI, some evidence points toward incongruity between subjective cognitive complaints and objective cognitive performance [43,44,45,46]. In a prospective longitudinal study that included 500 veterans with a TBI history (97% classified as mTBI), cognitive performance was found to be largely discrepant from their subjective complaints [47]. Veterans with an mTBI/TBI history reported persistent, greater problems with concentration, memory, decision-making, and slowed thinking relative to veterans without an mTBI/TBI history. However, overall, relatively few studies have yet examined subjective-objective cognitive congruity in veterans specifically with mTBI, while adjusting for psychiatric comorbidity [43,44,47]. Additionally, other research has demonstrated that veterans with mTBI objectively have lasting functional and cognitive changes that align with executive functioning difficulties seen on cognitive assessments [48,49,50,51]. Furthermore, veterans with PTSD and/or comorbid PTSD and mTBI/TBI have demonstrated executive dysfunction on objective assessments [52,53,54]. Among individuals with an mTBI/TBI history, the most subjectively reported cognitive complaints include memory as well as areas of executive functioning (EF), specifically working memory, inhibition, set shifting, planning and organizing, and task monitoring [46,55,56,57].

In regard to subjective cognitive concerns and childhood trauma, a prior study found a dose-response relationship between adverse childhood experiences and subjective cognitive decline in a sample of adults representative of the US population [58]. That is, as the number of adverse childhood experiences increased, the odds of endorsing subjective cognitive decline also increased, a finding consistent with additional studies [59,60]. No known studies have solely examined subjective-objective cognitive concordance among individuals with childhood trauma; however, naturally, early trauma may be a comorbid feature among individuals with other mental health conditions and backgrounds. For instance, individuals with more than four adverse childhood experiences are more likely to have a mental health diagnosis, substance use disorder, and housing problems [61]. Additionally, adverse childhood experiences also appear to be more prevalent among veterans [62,63], females [64], and minoritized racial/ethnic groups [61,64].

Although the majority of research appears to demonstrate limited subjective-objective cognitive concordance across populations, the value of exploring the contribution of subjective cognitive complaints should not be dismissed. The current study adds to the extant literature in several ways. First, relatively few studies have examined subjective-objective executive functioning concordance in mTBI groups while accounting for psychiatric and pre-existing factors. Although childhood trauma is relevant for the development of service-related PTSD, and PTSD is highly co-morbid with mTBI, no known studies have specifically accounted for childhood trauma while examining subjective-objective cognitive concordance in mTBI.

The congruity of subjective working memory complaints and objective working memory for individuals with an mTBI history remains unclear, especially for individuals with complex psychiatric comorbidity. Based on this lack of clarity and in the context that mTBI is associated with an increased risk of dementia [17,18,19,20,21], the current study plays a significant role in expanding the available literature. The current study aimed to further elucidate the congruity between subjective cognitive complaints and objective cognitive performance, with a focus on working memory, given that it has been a commonly reported complaint [6,36]. Additionally, the relationships between subjective and objective working memory were explored in relation to psychiatric symptoms (i.e., PTSD, depression, and sleep), mTBI severity, and childhood trauma. Finally, the extent of discrepancy in cognitive performance was evaluated.

## 2. Materials and Methods

The local Institutional Review Boards at Stanford University and the Palo Alto VA Healthcare System approved this study, and written informed consent was signed by all participants prior to data collection. During visits with a licensed clinical psychologist or supervised postdoctoral research fellow, participants completed self-report demographic and neuro-medical questionnaires as well as a comprehensive neuropsychological testing battery. Participants were recruited as part of a larger research study conducted at Stanford University and the Palo Alto VA. Seventy participants were in the entire cohort, and individuals who reported a history of a non-penetrating mTBI sustained during deployment were included in the current study (N = 35). mTBI history was verified through chart review as well as through clinical assessment. All study participants had a history of deployment-related mTBI sustained at least one year prior to enrollment.

### 2.1. Inclusion and Exclusion Criteria

The inclusion criteria were as follows: (1) A self-reported history of mTBI and/or PTSD (verified through chart review and a clinical assessment); (2) veteran receiving care at the VA Palo Alto Healthcare System; (3) ability to speak and read English; and (4) decision-making capacity for informed consent.

The exclusion criteria were as follows: (1) Diagnosis of a disorder affecting the central nervous system (e.g., seizure disorder, stroke); (2) diagnosis of dementia; (3) current psychosis; (4) history of substance use disorder within the past five years or illicit drug use within six months of study enrollment; (5) learning disabilities or attention-deficit hyperactivity disorder; (6) a penetrating TBI (as this can result in major anatomical and functional changes); and (7) anything preventing informed consent or participation (e.g., severe cognitive impairment), based on the clinical judgment of the provider (MM).

### 2.2. Evaluation and Clinical/Psychiatric Measures

Participants completed self-report questionnaires concerning demographic information and neuromedical history, including a detailed mTBI history [e.g., severity of mTBI, source of injury (blast versus blow to the head)]. Questionnaires were reviewed by the licensed provider, and a brief clinical interview was conducted to resolve any ambiguity. To measure symptoms of posttraumatic stress disorder, the PTSD Checklist Military Version (PCL-M) [65] was administered to all participants. The PCL-M is a 20-item self-report measure of PTSD symptoms experienced, measured on a Likert scale from 0 (not at all) to 4 (extremely), targeting symptoms over the past month. While the total score for this measure ranges from 0–68, for the purposes of the present study, we defined clinically significant symptoms of PTSD as scores of ≥45 points [66]. For relevant analyses, veterans were categorized into one of two groups based on the PCL-M total score: no PTSD (PCL-M score of 0–44) or PTSD (PCL-M score of 45 and above). To evaluate mTBI severity, a total score was developed based on positive endorsement of loss of consciousness, feeling dazed, loss of memory, being told they had experienced a concussion, and having a visible wound. Higher mTBI scores represent greater mTBI severity. The Beck Depression Inventory-II (BDI-II) is a 21-item self-report of depression symptoms, measured with a Likert scale (0 to 3), with higher scores representing greater depression [67]. The Pittsburgh Sleep Quality Index (PSQI) is a 19-item self-report questionnaire that measures the quality of sleep using a Likert scale (0 to 3), with higher scores representing worse sleep quality [68]. Childhood trauma was self-reported as the number of traumatic events experienced during childhood.

### 2.3. Subjective and Objective Neurocognitive Measures

The Behavioral Rating Inventory of Executive Functioning (BRIEF) is a self-report measure that assesses subjective executive functioning abilities. Questions are scored on a three-point Likert scale from 1 (never) to 3 (often) [69]. Higher scores represent greater self-reported executive difficulties. Responses are divided to create nine subscales assessing different components of executive functioning; for this study, only the working memory subscale was analyzed. In line with the administrative manual for this test, raw scores were converted to T-scores using the participants’ age. As part of a larger neurocognitive test battery, a number of measures were administered, including the Weschler Adult Intelligence Scale—Fourth Edition (WAIS-IV): Digit Span (DS) and Letter-Number Sequencing (LNS) subtests, which comprise the WAIS-IV working memory subtests. This analysis focused only on the WAIS-IV working memory subtests, as working memory is the most commonly reported complaint amongst veterans with a prior history of TBI. The WAIS-IV DS and LNS total scores were converted to standard scores to account for demographic variables (e.g., age). Standard scores for these two subtests were combined to create one composite working memory standard score.

### 2.4. Statistical Analysis

Statistical analyses were conducted using SPSS version 29. Summary statistics such as frequencies, mean (M), standard deviation (SD), and percentages were calculated for demographic information (e.g., age, education, gender, etc.). Data were screened for any violations of assumptions (e.g., normality, outliers, etc.) and were deemed appropriate for regression analyses.

For the primary aim, a linear regression analysis was used to examine whether subjective working memory (BRIEF working memory index; BRIEF WMI) was associated with objective cognitive performance (composite index score based on WAIS-IV DS and LNS; WAIS-IV WMI), accounting for PTSD, mTBI severity, and childhood trauma. Next, six correlations were utilized to examine associations between subjective and objective working memory and PTSD symptoms, depression symptoms, sleep quality, mTBI severity, and childhood trauma. Bonferroni corrections were utilized for the correlation analyses, with the adjusted *p*-value determined to be 0.0045, or *p* < 0.0001. Finally, the proportion of scores falling below, within, and higher than one standard deviation was calculated across the full sample and by PTSD group.

## 3. Results

### 3.1. Patient Characteristics

The sample included 35 veterans (11% female, aged 24 to 74 [M = 44.69, SD = 13.95]) who obtained 11 to 19 years of education (M = 14.63, SD = 1.78). Regarding mTBI characteristics, 29 veterans had a blast-related mTBI; 21 had an mTBI due to a concussive blow to the head. The sample had the following PCL-M-Total score (M = 45.31, SD = 20.36), with 18 veterans meeting criteria for clinically significant levels of PTSD symptoms (using a PCL-M cut-off of 45). The combined sample had the following BRIEF Working Memory score, M = 65.49, SD = 16.68, and the following WAIS-IV WMI score, M = 98.86, SD = 12.744. The majority of participants identified as male, were right-handed (n = 30, 85.7%), and were deployed (n = 35, 100%). Table 1 summarizes the demographic and clinical characteristics of the sample by PTSD symptom group. The PTSD symptom groups (no PTSD vs. PTSD) did not differ by any demographic variable (*ps* > 0.05) or by premorbid intellectual functioning (*p* = 0.66). For mTBI severity, scores ranged from zero to five. For childhood trauma, scores ranged from zero to six.

### 3.2. Primary Aims

#### Concordance of Subjective and Objective Working Memory, Accounting for mTBI Severity, PTSD, and Childhood Trauma

A linear regression analysis was conducted to assess whether subjective working memory and objective working memory might be related, controlling for PTSD, mTBI severity, and the number of childhood traumatic events. The model was not significant, F(3, 34) = 1.499, *p* = 0.234 (see Table 2 for results).

### 3.3. Additional Aims

#### Relationships between Subjective and Objective Working Memory with PTSD Symptoms, Depression Symptoms, Sleep Quality, mTBI Severity, and Childhood Trauma

Correlation analyses were conducted to assess PTSD, depression, and sleep symptoms in relation to subjective working memory (BRIEF WMI) and objective working memory (WAIS-IV WMI).

Regarding subjective working memory, the BRIEF WMI was significantly positively associated with PCL-M, *r*(34) = 0.75, *p* < 0.001, indicating that greater self-reports of working memory difficulties were related to increased reports of PTSD symptoms. Significance was maintained when Bonferroni-corrected (*p* < 0.0001). The BRIEF WMI was also significantly positively associated with the Beck Depression Inventory, even when Bonferroni-corrected, *r*(34) = 0.66, *p* < 0.0001, and the same relationship was seen with the Pittsburgh Sleep Quality Index, *r*(34) = 0.64, *p* < 0.0001. Respectively, this reflected that greater self-reports of working memory difficulties were associated with increased reports of depression symptoms as well as greater sleep problems. Subjective working memory was not significantly related to mTBI severity, *r*(34) = 0.27, *p* = 0.12. Subjective working memory was not significantly related to the number of childhood traumatic events, *r*(34) = 0.30, *p* = 0.08.

Regarding objective working memory, while not statistically significant, WAIS-IV WMI was negatively correlated with PCL-M, *r*(34) = −0.26, *p* = 0.13, which indicates that better objective working memory abilities were associated with fewer PTSD symptoms. Similarly, WAIS-IV WMI was negatively correlated with the Beck Depression Inventory, *r*(34) = −0.23, *p* = 0.19, as well as the Pittsburgh Sleep Quality Index, *r*(34) = −0.17, *p* = 0.31. While non-significant, the directionality points toward better objective working memory being related to fewer reported depression symptoms and worse sleep quality. Objective working memory was not significantly related to mTBI severity, *r*(34) = −0.28, *p* = 0.11. Objective working memory was significantly negatively related to the number of childhood traumatic events, *r*(34) = −0.43, *p* = 0.010, indicating that better objective working memory was related to fewer numbers of childhood traumatic events. However, significance was not maintained when adjusted using Bonferroni methods.

**Figure 1:**
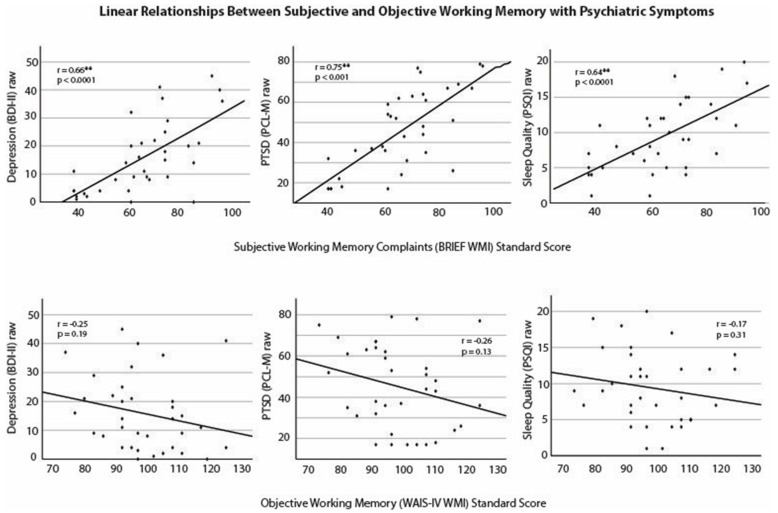
Note. Abbreviations: *BDI-II*, Beck Depression Inventory-II; *PCL-M*, PTSD Checklist Military Version; *PSQI*, Pittsburgh Sleep Quality Index; *PTSD*, post-traumatic stress disorder; *WAIS-IV WMI*, Weschler Adult Intelligence Scale—Fourth Edition, Working Memory Inventory; *BRIEF WMI*, Behavioral Rating Inventory of Executive Functioning, Working Memory Inventory.

Results that were sustained when using Bonferroni corrections (i.e., psychiatric symptoms with subjective working memory) are depicted in Figure 1, along with their related counterpart (i.e., psychiatric symptoms with objective working memory).

The proportion of scores falling below, within, and higher than one standard deviation was also examined in the full sample and by PTSD (see Table 3).

## 4. Discussion

The current results demonstrate limited congruity between subjective and objective working memory among individuals with a history of mTBI. Childhood trauma was a notable factor that contributed to both objective and subjective cognitive concerns; however, the relationship between childhood trauma and objective working memory did not withstand corrections. Greater subjective cognitive concerns were strongly associated with greater PTSD, depression, and sleep symptoms; objective cognitive functioning was not associated with any of these psychiatric concerns.

Overall, the full sample comprised individuals with intact objective working memory (i.e., within one standard deviation and above), despite the majority of all individuals (67%) endorsing significant subjective working memory complaints. Even with a relatively small sample size, 100% of the individuals who comprised the subsample group with an mTBI history and current severe PTSD endorsed significant subjective working memory complaints, despite the group performing well on objective tasks (i.e., 78% of the sample scored at least one standard deviation above the mean).

### 4.1. Concordance between Subjective and Objective Working Memory

Consistent with findings in other clinical populations, poor congruity between subjective working memory complaints and objective working memory was found among individuals with mTBI. Across various cognitive domains and clinical populations, several studies have demonstrated weak alignment between subjective cognitive complaints and objective cognitive data, such as in Parkinson’s disease, multiple sclerosis, and major depression [37,39,40]. Prior mTBI research found that a larger percentage of individuals with mTBI endorsed subjective cognitive concerns relative to non-mTBI individuals, despite overall similar performance between the two groups across cognitive domains [47]. For example, 70% of mTBI individuals endorsed attention and concentration symptoms, vs. 48% of non-mTBI individuals; however, both mTBI and non-mTBI groups demonstrated similar cognitive performance across attention and concentration tasks. As previously mentioned, the current study found that individuals with mTBI and current severe PTSD symptoms endorsed significant working memory concerns and performed well on objective working memory tasks. Thus, the present findings align with the recent mTBI literature indicating that subjective cognitive complaints are not well aligned with objective data.

### 4.2. Subjective, and Not Objective, Working Memory Was Related to PTSD, Depression, and Sleep Symptoms

There are several potential reasons for the observed discrepancy between subjective and objective cognition in mTBI. One relevant factor is that increased subjective cognitive concerns were strongly associated with greater PTSD and depression symptoms, as well as worse sleep quality, while objective cognitive functioning was not associated with any of the mentioned concerns. The current findings are thus in line with previous research that demonstrated the significant influence of psychiatric symptoms on perceived cognitive abilities [70]. It has previously been theorized that the incongruence between subjective and objective working memory is potentially due to response bias and generalized emotional distress rather than genuine executive dysfunction [46]. Within relevant TBI research, Caplan [71] found that greater affective distress (i.e., self-reported anxiety and depression) and sleep symptoms were related to increased subjective cognitive complaints, along with less time post-injury and older age, among individuals with cognitively intact abilities who had experienced a concussion. They also found that increased levels of affective distress and less time post-injury were related to increased cognitive discrepancy scores. Overall, the current study findings that subjective but not objective cognitive concerns are related to psychiatric distress are consistent with previous research and may also explain the subjective-objective cognitive incongruity. That is, it is possible that psychiatric and affective disturbances may be influencing subjective cognitive complaints and represent the “worried well”, rather than true cognitive dysfunction.

### 4.3. Possible Explanations for Subjective-Objective Cognitive Incongruity

However, there are additional explanations for subjective-objective cognitive incongruities that are potentially less invalidating of an individuals’ cognitive self-perceptions. It has previously been theorized that the discrepancy between subjective and objective difficulties may be due to the inaccuracy or inappropriateness of the traditional test instruments themselves. For instance, there may be an inability of objective measures to detect subtle cognitive changes (i.e., lacking sufficient sensitivity) [41]. In other words, certain assessments used may not be able to accurately capture individuals’ true cognitive abilities, especially for individuals with high premorbid abilities. Findings from a recent study by Karr and Hakun [72] support this notion, suggesting that high-functioning older adults may be experiencing neurological changes that do not align with neuropsychological scores. Additionally, early pathological mechanisms or biomarkers should also be considered in light of the study’s findings of subjective-objective cognitive discordance. In Alzheimer’s disease (AD), individuals with subjective cognitive decline and normal objective testing have been found to carry signature AD biomarkers such as cerebrospinal fluid abnormalities, increased brain atrophy, and increased hypometabolism in key regions affected by AD [73]. It may be that prior to observable objective difficulties, abnormal biological processes are indeed occurring that are only detected by individuals who are highly attuned to cognitive changes (e.g., individuals with depression). Alternative mechanisms, such as inflammation, may also interact with psychiatric symptoms and contribute to increased subjective cognitive complaints. For instance, among breast cancer survivors with depression, subjective cognitive concerns were reported, and objective cognitive difficulties were also observed; however, notably, survivors with both depression and immune dysregulation were more aware of depression-related cognitive deficits relative to other cancer survivors with depression [74].

In another vein, some widely used assessments may lack the most appropriate sociodemographic reference groups for increasingly globalized and multicultural societies. Heterogeneity across specific cognitive domains and tools may also be relevant. For instance, veterans with mTBI exhibited worse performance on objective inhibitory control measures based on a Go/No-Go task independent of psychiatric symptoms [75].

### 4.4. Childhood Trauma and Subjective Cognitive Concerns

There is growing evidence that pre-service trauma, such as childhood abuse, renders an increased risk for developing PTSD, even after accounting for combat exposure [14]. Additionally, research points to adverse childhood experiences being more prevalent among veterans [62,63]. To our knowledge, this is the first study to specifically account for childhood trauma while examining subjective-objective cognitive concordance, within the context of PTSD and mTBI among veterans. We found that childhood trauma was a significant predictor of subjective cognitive concerns. It was also found that an increased number of childhood traumatic events was related to worse objective working memory, which did not withstand Bonferroni corrections. Previously, multiple studies have found that individuals with adverse childhood experiences exhibited significantly greater odds of reporting subjective cognitive decline as adults [58,59,60]. In regard to objective working memory, among healthy adults, individuals with a history of childhood trauma demonstrated poorer cognitive performance, particularly in working memory [76,77]. The current study thus aligns with results derived from the general adult population. Overall, the inclusion of childhood trauma provides further support regarding the importance of psychological stability and mental health prior to wartime exposure. That is, it solidifies the notion that it is crucial to identify and provide support for individuals who might be at heightened risk for poor outcomes following service and combat exposure [78].

### 4.5. Importance of Continued Examination of Subjective Cognitive Complaints

Despite the subjective-objective cognitive incongruity, subjective cognitive assessment continues to be clinically vital. Veterans, in particular, represent an aging population, with an average age of approximately 65 across all veterans. Thus, the veteran population, and specifically this sample, represents an at-risk group for developing future age-related neurocognitive disorders. Additionally, the intersectionality of psychiatric and vascular comorbidity also places veterans at increased cognitive disorder risk, given high rates of psychiatric and vascular conditions relative to the general population [79].

Though the diagnostic validity of subjective cognitive complaints for cognitive impairment has been questioned [32,33,34], it is possible that subjective cognitive decline may flag or represent an early dementia sign [27,28,29,30,31]. Several meta-analyses have found that subjective cognitive concerns are predictive of future cognitive decline, while objective cognitive impairment on objective measures was not evidenced [41,80,81]. Recent research has thus been working on developing models to characterize the progression from subjective cognitive decline to mild cognitive impairment or dementia [82]. Specifically among veterans with mTBI, research has also found that subjective cognitive concerns (i.e., prospective and retrospective memory dysfunction) were associated with decreased cortical thickness in specific brain regions, while objective deficits were not revealed on traditional assessments [83]. Additionally, in a recent study among healthy adults, baseline Alzheimer’s biomarkers (e.g., CSF markers, hippocampal volume) predicted self-reported cognitive decline over time, based on reports from both the participant and an informant [84]. The authors thus suggest that subjective cognitive complaints are clinically useful in monitoring subtle cognitive changes and may flag individuals for additional diagnostic procedures (e.g., lumbar puncture, PET scans, etc.). Overall, future studies may benefit from developing more sophisticated models and adding measurements of neurobiological substrates to better elucidate cognitive performance rather than analyzing subject cognitive complaints and traditional cognitive assessments alone.

### 4.6. Clinical Implications

It is helpful for clinicians to be aware of the subjective-objective cognitive incongruity as well as the factors that can influence subjective cognitive concerns. The presence of greater subjective cognitive concerns relative to objective cognitive data may flag the possibility of the presence of psychiatric distress, with intervention focused on targeting depression and anxiety symptoms. Potential treatment recommendations may be geared toward mood or cognition, or both. For example, if a veteran reported cognitive complaints and has a remote history (i.e., longer than two or three years) of TBI and emotional distress (from PTSD), primary recommendations can be psychological interventions, with secondary approaches being cognitive rehabilitation. By addressing factors that potentially impact subjective complaints, the alleviation of concerns can be addressed and complaints can potentially decrease. Intervention among individuals with comorbid PTSD and psychiatric concerns is especially important, given that subjective cognitive difficulties have been related to a decreased ability to perform activities of daily living as well as a poorer quality of life [85,86]. Thus, subjective cognitive decline must continue to be assessed and thoughtfully considered by health care providers.

Additionally, early life experiences are recognized to strongly contribute to one’s physical, mental, and emotional well-being throughout the lifespan, such that if early deleterious events occur, the consequences may potentially be lifelong (e.g., increased risk of mental health conditions [61]). The study findings surrounding childhood trauma support the notion of developing programs and policies that build resilience in young individuals facing adversity.

### 4.7. Limitations

Regarding limitations, this study included a relatively small number of participants with a predominantly white, male sample. Future studies should investigate this area with a larger sample with diverse gender and ethnic representation to increase generalizability. Notably, the current study utilized a cross-sectional design, which precludes causational understanding from the findings; more rigorous future research, including longitudinal designs, is thus warranted. While the parent study collected neuroimaging data (e.g., diffusion tensor imaging), there was an inadequate sample size to run analyses of the present study’s variables of interest (n < 10). Future studies that incorporate neuroimaging would help to better understand the subjective-objective cognitive discordance and provide insight into how cerebral changes may impact subjective cognitive concerns despite objectively normal performance. Most of the participants also experienced blast or blow-based head injuries, which are characteristic of most head injuries for veterans deployed to Afghanistan and Iraq [87], although this does not generalize to other injuries. Future research should continue to investigate the role of different types of TBI (i.e., accident/crash, fall, fragment wound) as well as differences in the severity of TBI (i.e., concussion, vision concussion, vision impairments, memory lapses) on both subjective and objective working memory. Lastly, there should be a continuation of current investigations into how PTSD diagnosis, as well as a history of brain injuries, might express differing responses and congruity of cognition aside from working memory.

## 5. Conclusions

Overall, the current study validates previous research, with limited congruity found between subjective working memory concerns and objective working memory difficulties in individuals with an mTBI history. Despite the subjective-objective working memory incongruity found within this sample, there remains clinical value in assessing subjective cognitive complaints. The strong relationship with psychiatric and affective distress in daily life can help to flag and focus treatment interventions. Additionally, burgeoning research has pointed to the potential for subjective cognitive complaints to detect a future cognitive disorder.

The present study also adds novel information by integrating childhood trauma, as this is the first known study to account for childhood trauma while examining subjective-objective cognitive concordance within the context of PTSD and mTBI. Childhood trauma was a key factor in both subjective and objective cognitive concerns, thus supporting the importance of identifying and counseling individuals who may be at higher risk for poor outcomes following service.

## Figures and Tables

**Table 1:** Demographic and clinical characteristics across the full sample (N = 35).

	M (SD) or n (%)
Age	44 (14)
Gender	
Male	31 (87%)
Female	4 (11%)
Race	
White	24 (69%)
Black	4 (11%)
Asian	2 (6%)
American Indian/Alaska Native	1 (3%)
Native Hawaiian/Pacific Islander	2 (6%)
Prefers not to answer	1 (3%)
Other	1 (3%)
Ethnicity	
Hispanic or Latino	25 (72%)
Non-Hispanic	8 (23%)
Unknown	2 (6%)
Education (years)	14 (2)
HS graduate or less	3 (9%)
Some college	18 (51%)
College graduate	12 (34%)
Post college	2 (6%)
Handedness	
Right	30 (86%)
Left	3 (9%)
Amidexterous	2 (6%)
PTSD ^a^	
No PTSD	18 (51%)
PTSD	17 (49%)
Depression (BDI-II)	15 (13)
Sleep Quality	9 (5)
PTSD Checklist	45 (20)
WAIS WMI	98 (13)
BRIEF WMI	65 (17)

Note: Due to rounding errors, percentages may not equal 100%. Abbreviations: *BDI-II*, Beck Depression Inventory-II; *BRIEF WMI*, Behavioral Rating Inventory of Executive Functioning, Working Memory Inventory; *mTBI*, mild traumatic brain injury; *PSQI*, Pittsburgh Sleep Quality Index; *PCL-M*, PTSD Checklist Military Version; *PTSD*, post-traumatic stress disorder; *WAIS-IV WMI*, Weschler Adult Intelligence Scale—Fourth Edition, Working Memory Inventory. ^a^ Categorized based on PCL-M total scores: No PTSD (PCL-M score of 0–44); PTSD (PCL-M score of 45 and above).

**Table 2:** Results for linear regression model examining subjective and objective working memory, controlling for PTSD, mTBI severity, and childhood trauma.

Variable	B	SE	β	t	Sig.
(Intercept)	1.33	18.80		0.07	0.94
Objective working memory (WAIS WMI)	0.30	0.16	0.23	1.85	0.07
PTSD (PCL-M)	0.64	0.10	0.78	6.49	<0.0001 **
mTBI severity	−0.60	1.16	−0.07	−0.52	0.61
Childhood trauma	3.18	1.33	0.30	2.40	0.02 *
		R	R-sq.	F	Sig.
DV: Subjective working memory (BRIEF WMI)		0.80	0.53	13.14	<0.0001 **

Note. Abbreviations: *BRIEF WMI*, Behavioral Rating Inventory of Executive Functioning, Working Memory Inventory; *mTBI*, mild traumatic brain injury; *PCL-M*, PTSD Checklist Military Version; *PTSD*, post-traumatic stress disorder; *WAIS-IV WMI*, Weschler Adult Intelligence Scale—Fourth Edition. * *p* < 0.05. ** *p* < 0.001.

**Table 3:** Proportion of scores equal to or exceeding one standard deviation on objective and subjective measures.

		Full mTBI Sample (N = 35)	mTBI + PTSD ^a^ (n = 18)	mTBI + no PTSD ^a^ (n = 17)
Objective Working Memory		n (%)	n (%)	n (%)
WAIS-IV WMI				
Low		0 (0%)	0 (0%)	0 (0%)
Normal		5 (14%)	4 (22%)	1 (6%)
High		30 (86%)	14 (78%)	16 (94%)
Subjective Working Memory				
BRIEF WMI				
Low		3 (9%)	0 (0%)	3 (18%)
Normal		8 (23%)	0 (0%)	8 (47%)
High		24 (69%)	18 (100%)	6 (35%)

Abbreviations: *mTBI*, mild traumatic brain injury; *PTSD*, post-traumatic stress disorder; *WAIS-IV WMI*, Weschler Adult Intelligence Scale—Fourth Edition, Working Memory Inventory; *BRIEF WMI*, Behavioral Rating Inventory of Executive Functioning, Working Memory Inventory. ^a^ Categorized based on PCL-M total scores: Minimal PTSD (PCL-M score of 0–44); severe PTSD (PCL-M score of 45 and above).

## Data Availability

Raw data were generated at Stanford University and Palo Alto VA. Derived data supporting the findings of this study are available from the corresponding author (MM) upon request.

## References

[1] Terrio H., Brenner L.A., Ivins B.J., Cho J.M., Helmick K., Schwab K., Scally K., Bretthauer R., Warden D. (2009). Traumatic brain injury screening: Preliminary findings in a US Army Brigade Combat Team. J. Head Trauma Rehabil..

[2] Hoge C.W., McGurk D., Thomas J.L., Cox A.L., Engel C.C., Castro C.A. (2008). Mild Traumatic brain injury in U.S. soldiers returning from Iraq. N. Engl. J. Med..

[3] Schneiderman A.I., Braver E.R., Kang H.K. (2008). Understanding sequelae of injury mechanisms and mild traumatic brain injury incurred during the conflicts in Iraq and afghanistan: Persistent postconcussive symptoms and posttraumatic stress disorder. Am. J. Epidemiol..

[4] Lindquist L.K., Love H.C., Elbogen E.B. (2017). Traumatic Brain Injury in Iraq and Afghanistan Veterans: New Results From a National Random Sample Study. J. Neuropsychiatry.

[5] Carlson K.F., Kehle S.M., Meis L.A., Greer N., MacDonald R., Rutks I., Sayer N.A., Dobscha S.K., Wilt T.J. (2011). Prevalence, assessment, and treatment of mild traumatic brain injury and posttraumatic stress disorder: A systematic review of the evidence. J. Head Trauma Rehabil.

[6] Dieter J.N., Engel S.D. (2019). Traumatic Brain Injury and Posttraumatic Stress Disorder: Comorbid Consequences of War. Neurosci. Insights.

[7] Hayes J.P. (2019). PTSD and TBI Comorbidity. PTSD Res. Q..

[8] Tanev K.S., Pentel K.Z., Kredlow M.A., Charney M.E. (2014). PTSD and TBI co-morbidity: Scope, clinical presentation and treatment options. Brain Inj..

[9] Jackson C.E., Green J.D., Bovin M.J., Vasterling J.J., Holowka D.W., Ranganathan G., Rosen R.C., Keane T.M., Marx B.P. (2016). Mild Traumatic Brain Injury, PTSD, and Psychosocial Functioning Among Male and Female U.S. OEF/OIF Veterans. J. Trauma. Stress..

[10] Pietrzak R.H., Johnson D.C., Goldstein M.B., Malley J.C., Southwick S.M. (2009). Posttraumatic stress disorder mediates the relationship between mild traumatic brain injury and health and psychosocial functioning in veterans of operations enduring freedom and Iraqi freedom. J. Nerv. Ment. Dis..

[11] Vasterling J.J., Brailey K., Proctor S.P., Kane R., Heeren T., Franz M. (2012). Neuropsychological outcomes of mild traumatic brain injury, post-traumatic stress disorder and depression in Iraq-deployed US Army soldiers. Br. J. Psychiatry.

[12] Owens G.P., Dashevsky B., Chard K.M., Mohamed S., Haji U., Heppner P.S., Baker D.G. (2009). The Relationship Between Childhood Trauma, Combat Exposure, and Posttraumatic Stress Disorder in Male Veterans. Mil. Psychol..

[13] Zaidi L.Y., Foy D.W. (1994). Childhood abuse experiences and combat-related PTSD. J. Trauma. Stress..

[14] Van Voorhees E.E., Dedert E.A., Calhoun P.S., Brancu M., Runnals J., Beckham J.C., VA Mid-Atlantic MIRECC Workgroup (2012). Childhood trauma exposure in Iraq and Afghanistan war era veterans: Implications for posttraumatic stress disorder symptoms and adult functional social support. Child Abuse Negl..

[15] Gillespie C.F., Phifer J., Bradley B., Ressler K.J. (2009). Risk and resilience: Genetic and environmental influences on development of the stress response. Depress. Anxiety.

[16] Koenen K.C. (2006). Developmental epidemiology of PTSD: Self-regulation as a central mechanism. Ann N. Y. Acad Sci..

[17] Snowden T.M., Hinde A.K., Reid H.M., Christie B.R. (2020). Does Mild Traumatic Brain Injury Increase the Risk for Dementia? A Systematic Review and Meta-Analysis. J. Alzheimer’s Dis..

[18] Lee Y.-K., Hou S.-W., Lee C.-C., Hsu C.-Y., Huang Y.-S., Su Y.-C. (2013). Increased risk of dementia in patients with mild traumatic brain injury: A nationwide cohort study. PLoS ONE.

[19] Barnes D.E., Byers A.L., Gardner R.C., Seal K.H., Boscardin W.J., Yaffe K. (2018). Association of Mild Traumatic Brain Injury With and Without Loss of Consciousness With Dementia in US Military Veterans. JAMA Neurol..

[20] Raza Z., Hussain S.F., Ftouni S., Spitz G., Caplin N., Foster R.G., Gomes R.S.M. (2021). Dementia in military and veteran populations: A review of risk factors-traumatic brain injury, post-traumatic stress disorder, deployment, and sleep. Mil. Med. Res..

[21] Pattinson C.L., Gill J.M. (2018). Risk of dementia after TBI—A cause of growing concern. Nat. Rev. Neurol..

[22] Günak M.M., Billings J., Carratu E., Marchant N.L., Favarato G., Orgeta V. (2020). Post-traumatic stress disorder as a risk factor for dementia: Systematic review and meta-analysis. Br. J. Psychiatry.

[23] Yaffe K., Vittinghoff E., Lindquist K., Barnes D., Covinsky K.E., Neylan T., Kluse M., Marmar C. (2010). Posttraumatic stress disorder and risk of dementia among US veterans. Arch. Gen. Psychiatry.

[24] Xie Z., Li M., Sun H., Zhou C., Fu C., Wang Q., Dong C., Hao W., Zhen X., Zhu D. (2023). Childhood, adulthood, and cumulative traumatic events experienced from childhood to adulthood and dementia risk. J. Public Health.

[25] Abouelmagd M.E., AbdelMeseh M., Elrosasy A., Eldeeb H.A., Nabil Y. (2024). Adverse childhood experiences and risk of late-life dementia: A systematic review and meta-analysis. Soc. Psychiatry Psychiatr Epidemiol..

[26] Vespa J. (2023). Aging Veterans: America’s Veteran Population in Later Life. American Community Survey Reports.

[27] Wang X.-T., Wang Z.-T., Hu H.-Y., Qu Y., Wang M., Shen X.-N., Xu W., Dong Q., Tan L., Yu J.-T. (2021). Association of Subjective Cognitive Decline with Risk of Cognitive Impairment and Dementia: A Systematic Review and Meta-Analysis of Prospective Longitudinal Studies. J. Prev. Alzheimer’s Dis..

[28] Ávila-Villanueva M., Rebollo-Vázquez A., de León J.M.R.-S., Valentí M., Medina M., Fernández-Blázquez M.A. (2016). Clinical Relevance of Specific Cognitive Complaints in Determining Mild Cognitive Impairment from Cognitively Normal States in a Study of Healthy Elderly Controls. Front. Aging Neurosci..

[29] Dubois B., Hampel H., Feldman H.H., Scheltens P., Aisen P., Andrieu S., Bakardjian H., Benali H., Bertram L., Blennow K. (2016). Preclinical Alzheimer’s disease: Definition, natural history, and diagnostic criteria. Alzheimers Dement.

[30] Jessen F., Amariglio R.E., Van Boxtel M., Breteler M., Ceccaldi M., Chételat G., Dubois B., Dufouil C., Ellis K.A., Van Der Flier W.M. (2014). A conceptual framework for research on subjective cognitive decline in preclinical Alzheimer’s disease. Alzheimers Dement.

[31] Jessen F., Amariglio R.E., Buckley R.F., van der Flier W.M., Han Y., Molinuevo J.L., Rabin L., Rentz D.M., Rodriguez-Gomez O., Saykin A.J. (2020). The characterisation of subjective cognitive decline. Lancet Neurol..

[32] Jonker C., Geerlings M.I., Schmand B. (2000). Are memory complaints predictive for dementia? A review of clinical and population-based studies. Int. J. Geriatr. Psychiatry.

[33] Reid L.M., MacLullich A.M. (2006). Subjective memory complaints and cognitive impairment in older people. Dement. Geriatr. Cogn. Disord..

[34] Stewart R. (2012). Mild cognitive impairment—The continuing challenge of its “real-world” detection and diagnosis. Arch Med. Res..

[35] Amick M.M., Meterko M., Fortier C.B., Fonda J.R., Milberg W.P., McGlinchey R.E. (2018). The Deployment Trauma Phenotype and Employment Status in Veterans of the Wars in Iraq and Afghanistan. J. Head. Trauma. Rehabil..

[36] Seal K.H., Bertenthal D., Samuelson K., Maguen S., Kumar S., Vasterling J.J. (2016). Association between mild traumatic brain injury and mental health problems and self-reported cognitive dysfunction in Iraq and Afghanistan Veterans. J. Rehabil. Res. Dev..

[37] Purri R., Brennan L., Rick J., Xie S.X., Deck B.L., Chahine L.M., Dahodwala N., Chen-Plotkin A., Duda J.E., Morley J.F. (2020). Subjective Cognitive Complaint in Parkinson’s Disease Patients With Normal Cognition: Canary in the Coal Mine?. Mov. Disord.

[38] Siciliano M., Trojano L., De Micco R., Sant’elia V., Giordano A., Russo A., Passamonti L., Tedeschi G., Chiorri C., Tessitore A. (2021). Correlates of the discrepancy between objective and subjective cognitive functioning in non-demented patients with Parkinson’s disease. Neurol.

[39] Thomas G.A., Riegler K.E., Bradson M.L., O’shea D.U., Arnett P.A. (2023). Relationship between subjective report and objective assessment of neurocognitive functioning in persons with multiple sclerosis. J. Int. Neuropsychol. Soc..

[40] Srisurapanont M., Suttajit S., Eurviriyanukul K., Varnado P. (2017). Discrepancy between objective and subjective cognition in adults with major depressive disorder. Sci. Rep..

[41] Burmester B., Leathem J., Merrick P. (2016). Subjective Cognitive Complaints and Objective Cognitive Function in Aging: A Systematic Review and Meta-Analysis of Recent Cross-Sectional Findings. Neuropsychol. Rev..

[42] Crumley J.J., Stetler C.A., Horhota M. (2014). Examining the relationship between subjective and objective memory performance in older adults: A meta-analysis. Psychol. Aging.

[43] Merritt V.C., Jurick S.M., Crocker L.D., Sullan M.J., Sakamoto M.S., Davey D.K., Hoffman S.N., Keller A.V., Jak A.J. (2020). Associations Between Multiple Remote Mild TBIs and Objective Neuropsychological Functioning and Subjective Symptoms in Combat-Exposed Veterans. Arch. Clin. Neuropsychol..

[44] Neale A.C., Aase D.M., Soble J.R., Baker J.C., Phan K.L. (2022). Disentangling subjective symptom complaints and objective cognitive performance in veterans: Impact of posttraumatic stress disorder and lifetime traumatic brain injury burden. Appl. Neuropsychol. Adult.

[45] Scott T., Spellman J., Walker N., Rivera J., Waltzman D., Mcnerney M., Madore M. (2020). A-09 The Relationship Between Subjective Cognitive Complaints, Depression, and Executive Functioning in TBI Veterans. Arch. Clin. Neuropsychol..

[46] Shwartz S.K., Roper B.L., Arentsen T.J., Crouse E.M., Adler M.C. (2020). The Behavior Rating Inventory of Executive Function(R)-Adult Version is Related to Emotional Distress, Not Executive Dysfunction, in a Veteran Sample. Arch Clin. Neuropsychol..

[47] Donnelly K., Donnelly J.P., Warner G.C., Kittleson C.J., King P.R. (2017). Longitudinal study of objective and subjective cognitive performance and psychological distress in OEF/OIF Veterans with and without traumatic brain injury. Clin. Neuropsychol..

[48] Clark J.M.R., Mahmood Z., Jak A.J., Huckans M., O’Neil M.E., Roost M.S., Williams R.M., Turner A.P., Pagulayan K.F., Storzbach D. (2022). Neuropsychological Performance and Functional Capacity Following Mild Traumatic Brain Injury in Veterans. J. Head. Trauma. Rehabil..

[49] Clausen A.N., Bouchard H.C., Workgroup V.M.-A.M., Welsh-Bohmer K.A., Morey R.A. (2021). Assessment of Neuropsychological Function in Veterans With Blast-Related Mild Traumatic Brain Injury and Subconcussive Blast Exposure. Front. Psychol..

[50] Geuze E., Vermetten E., de Kloet C.S., Hijman R., Westenberg H.G.M. (2009). Neuropsychological performance is related to current social and occupational functioning in veterans with posttraumatic stress disorder. Depress. Anxiety.

[51] Koso M., Hansen S. (2006). Executive function and memory in posttraumatic stress disorder: A study of Bosnian war veterans. Eur. Psychiatry.

[52] Esopenko C., de Souza N.L.M., Jia Y., Parrott J.S., Merkley T.L., Dennis E.L., Hillary F.G., Velez C.M., Cooper D.B., Kennedy J. (2022). Latent Neuropsychological Profiles to Discriminate Mild Traumatic Brain Injury and Posttraumatic Stress Disorder in Active-Duty Service Members. J. Head. Trauma. Rehabil..

[53] Jagger-Rickels A., Rothlein D., Stumps A., Evans T.C., Bernstein J., Milberg W., McGlinchey R., DeGutis J., Esterman M. (2022). An executive function subtype of PTSD with unique neural markers and clinical trajectories. Transl. Psychiatry.

[54] Jurick S.M., Crocker L.D., Merritt V.C., Sanderson-Cimino M.E., Keller A.V., Glassman L.H., Twamley E.W., Rodgers C.S., Schiehser D.M., Aupperle R.L. (2021). Independent and Synergistic Associations Between TBI Characteristics and PTSD Symptom Clusters on Cognitive Performance and Postconcussive Symptoms in Iraq and Afghanistan Veterans. J. Neuropsychiatry Clin. Neurosci..

[55] Karr J.E., Rau H.K., Shofer J.B., Hendrickson R.C., Peskind E.R., Pagulayan K.F. (2019). Variables associated with subjective cognitive change among Iraq and Afghanistan war Veterans with blast-related mild traumatic brain injury. J. Clin. Exp. Neuropsychol..

[56] Mattson E.K., Nelson N.W., Sponheim S.R., Disner S.G. (2019). The Impact of PTSD and mTBI on the Relationship between Subjective and Objective Cognitive Deficits in Combat-Exposed Veterans. Neuropsychology.

[57] Pagulayan K.F., Petrie E.C., Cook D.G., Hendrickson R.C., Rau H., Reilly M., Mayer C., Meabon J.S., Raskind M.A., Peskind E.R. (2020). Effect of blast-related mTBI on the working memory system: A resting state fMRI study. Brain Imaging Behav..

[58] Brown M.J., Kaur A., James T., Avalos C., Addo P.N.O., Crouch E., Hill N.L. (2022). Adverse Childhood Experiences and Subjective Cognitive Decline in the US. J. Appl. Gerontol..

[59] Terry R.M., Schiffmacher S.E., Dutcher A.A., Croff J.M., Jelley M.J., Hartwell M.L. (2023). Adverse childhood experience categories and subjective cognitive decline in adulthood: An analysis of the Behavioral Risk Factor Surveillance System. J. Am. Osteopat. Assoc..

[60] Baiden P., Cassidy J., Panisch L.S., LaBrenz C.A., Onyeaka H.K. (2021). Association of adverse childhood experiences with subjective cognitive decline in adulthood: Findings from a population-based study. Aging Ment. Health.

[61] Madigan S., Deneault A., Racine N., Park J., Thiemann R., Zhu J., Dimitropoulos G., Williamson T., Fearon P., Cénat J.M. (2023). Adverse childhood experiences: A meta-analysis of prevalence and moderators among half a million adults in 206 studies. World Psychiatry.

[62] Blosnich J.R., Dichter M.E., Cerulli C., Batten S.V., Bossarte R.M. (2014). Disparities in adverse childhood experiences among individuals with a history of military service. JAMA Psychiatry.

[63] Aronson K.R., Perkins D.F., Morgan N.R., Bleser J.A., Vogt D., Copeland L.A., Finley E.P., Gilman C.L. (2020). The impact of adverse childhood experiences (ACEs) and combat exposure on mental health conditions among new post-9/11 veterans. Psychol Trauma.

[64] Giano Z., Wheeler D.L., Hubach R.D. (2020). The frequencies and disparities of adverse childhood experiences in the U.S. BMC Public Health.

[65] Weathers F., Huska J., Keane T. (1991). The PTSD Checklist Military Version (PCL-M).

[66] Blanchard E.B., Jones-Alexander J., Buckley T.C., Forneris C.A. (1996). Psychometric properties of the PTSD checklist (PCL). Behav. Res. Ther..

[67] Beck A.T., Steer R.A., Brown G.K. (1996). Manual for the Beck Depression Inventory-II.

[68] Buysse D.J., Reynolds C.F., Monk T.H., Hoch C.C., Yeager A.L., Kupfer D.J. (1991). Quantification of subjective sleep quality in healthy elderly men and women using the pittsburgh sleep quality index (PSQI). Sleep.

[69] Gioia G.A., Isquith P.K., Guy S.C., Kenworthy L. (2000). The Behavior Rating Inventory of Executive Function.

[70] Hill N.L., Mogle J., Wion R., Munoz E., DePasquale N., Yevchak A.M., Parisi J.M. (2016). Subjective Cognitive Impairment and Affective Symptoms: A Systematic Review. Gerontologist.

[71] Caplan B., Bogner J., Brenner L., Malec J., Hromas G.A., Houck Z.M., Bauer R.M. (2021). Making a difference: Affective distress explains discrepancy between objective and subjective cognitive functioning after mild traumatic brain injury. J. Head Trauma Rehabil..

[72] Karr J.E., Hakun J.G., Elbich D.B., Pinheiro C.N., Schmitt F.A., Segerstrom S.C. (2024). Detecting cognitive decline in high-functioning older adults: The relationship between subjective cognitive concerns, frequency of high neuropsychological test scores, and the frontoparietal control network. J. Int. Neuropsychol. Soc..

[73] Lista S., Molinuevo J.L., Cavedo E., Rami L., Amouyel P., Teipel S.J., Garaci F., Toschi N., Habert M.-O., Blennow K. (2015). Evolving Evidence for the Value of Neuroimaging Methods and Biological Markers in Subjects Categorized with Subjective Cognitive Decline. J. Alzheimer’s Dis..

[74] Madison A.A., Andridge R., Kantaras A.H., Renna M.E., Bennett J.M., Alfano C.M., Povoski S.P., Agnese D.M., Lustberg M., Wesolowski R. (2023). Depression, Inflammation, and Intestinal Permeability: Associations with Subjective and Objective Cognitive Functioning throughout Breast Cancer Survivorship. Cancers.

[75] Holiday K.A., Clark A.L., Merritt V.C., Nakhla M.Z., Sorg S., Delano-Wood L., Schiehser D.M. (2020). Response inhibition in Veterans with a history of mild traumatic brain injury: The role of self-reported complaints in objective performance. J. Clin. Exp. Neuropsychol..

[76] Majer M., Nater U.M., Lin J.-M.S., Capuron L., Reeves W.C. (2010). Association of childhood trauma with cognitive function in healthy adults: A pilot study. BMC Neurol..

[77] Dodaj A., Krajina M., Sesar K., Šimić N. (2017). The Effects of Maltreatment in Childhood on Working Memory Capacity in Adulthood. Eur. J. Psychol..

[78] McGlinchey R.E., Milberg W.P., Fonda J.R., Fortier C.B. (2017). A methodology for assessing deployment trauma and its consequences in OEF/OIF/OND veterans: The TRACTS longitudinal prospective cohort study. Int. J. Methods Psychiatr. Res..

[79] Dyball D., Evans S., Boos C.J., Stevelink S.A.M., Fear N.T. (2019). The association between PTSD and cardiovascular disease and its risk factors in male veterans of the Iraq/Afghanistan conflicts: A systematic review. Int. Rev. Psychiatry.

[80] Mitchell A.J., Beaumont H., Ferguson D., Yadegarfar M., Stubbs B. (2014). Risk of dementia and mild cognitive impairment in older people with subjective memory complaints: Meta-analysis. Acta Psychiatr. Scand..

[81] Pike K.E., Cavuoto M.G., Li L., Wright B.J., Kinsella G.J. (2022). Subjective Cognitive Decline: Level of Risk for Future Dementia and Mild Cognitive Impairment, a Meta-Analysis of Longitudinal Studies. Neuropsychol. Rev..

[82] An R., Gao Y., Huang X., Yang Y., Yang C., Wan Q. (2024). Predictors of progression from subjective cognitive decline to objective cognitive impairment: A systematic review and meta-analysis of longitudinal studies. Int. J. Nurs. Stud..

[83] Ly M.T., Merritt V.C., Ozturk E.D., Clark A.L., Hanson K.L., Delano-Wood L.M., Sorg S.F. (2023). Subjective memory complaints are associated with decreased cortical thickness in Veterans with histories of mild traumatic brain injury. Clin. Neuropsychol..

[84] Levine T.F., Dessenberger S.J., Allison S.L., Head D., Initiative T.A.D.N. (2024). Alzheimer disease biomarkers are associated with decline in subjective memory, attention, and spatial navigation ability in clinically normal adults. J. Int. Neuropsychol. Soc..

[85] Hill N.L., McDermott C., Mogle J., Munoz E., DePasquale N., Wion R., Whitaker E. (2017). Subjective cognitive impairment and quality of life: A systematic review. Int. Psychogeriatr..

[86] Roehr S., Luck T., Pabst A., Bickel H., König H.-H., Lühmann D., Fuchs A., Wolfsgruber S., Wiese B., Weyerer S. (2017). Subjective cognitive decline is longitudinally associated with lower health-related quality of life. Int. Psychogeriatr..

[87] Wojcik B.E., Stein C.R., Bagg K., Humphrey R.J., Orosco J. (2010). Traumatic brain injury hospitalizations of U.S. army soldiers deployed to afghanistan and Iraq. Am. J. Prev. Med..

